# Supplementary material for: Evaluation and deployment of isotype-specific salivary antibody assays for detecting previous SARS-CoV-2 infection in children and adults
Source: Commun Med (Lond). 2023 Mar 15;3:37. doi: 10.1038/s43856-023-00264-2 (PMC10016188; doi:10.1038/s43856-023-00264-2)
Supplement: Supplementary file 7 — Description of Additional Supplementary Files [file 43856_2023_264_MOESM7_ESM.pdf]

Description of Additional Supplementary Files

File Name: Supplementary Data 1
Description:
Data underlying Figure 2. Sheet 1 contains underlying data for dotplots shown in panels a to f, antibody level (normalised OD) by antigen (N-protein/RBD/spike), isotype (IgA/IgG) and sample group (pre-pandemic/PCR confirmed). Sheets 2 to 7 contain underlying data for ROC curves given in panels g to i, organised by antigen and isotype.

File Name: Supplementary Data 2

Description:
Data underlying Figure 3. Sheet 1 contains underlying data for panel a, antibody level (normalised OD) by antigen (N-protein/RBD/spike), isotype (IgA/IgG) and sample group (pre-pandemic/PCR confirmed). Sheet 2 contains underlying data for panel b, organised by assay combination (mean and each CV fold score).

File Name: Supplementary Data 3

Description:
Data underlying Figure 4. Antibody level (normalised OD) by antigen (N-protein/RBD/spike), isotype (IgA/IgG), sample group (pre-pandemic/PCR confirmed) and sample type (saliva/serum). Each row is a paired (saliva and serum) case.

File Name: Supplementary Data 4
Description:
Data underlying Figure 5. Sheets 1 to 4 contain data underlying panels a to d respectively, organised by antigen (N-protein/spike), isotype (IgA/IgG/IgA or IgG) and sample group (PCR positive cases/PCR negative contacts).
