# Supplementary material for: Evaluation and deployment of isotype-specific salivary antibody assays for detecting previous SARS-CoV-2 infection in children and adults
Source: Commun Med (Lond). 2023 Mar 15;3:37. doi: 10.1038/s43856-023-00264-2 (PMC10016188; doi:10.1038/s43856-023-00264-2)
Supplement: Supplementary file 8 — Reporting Summary [file 43856_2023_264_MOESM8_ESM.pdf]

nature portfolio

Corresponding author(s): Amy Thomas

Last updated by author(s): Jan 25, 2023

Reporting Summary

Nature Portfolio wishes to improve the reproducibility of the work that we publish. This form provides structure for consistency and transparency in reporting. For further information on Nature Portfolio policies, see our Editorial Policies and the Editorial Policy Checklist.

Statistics

For all statistical analyses, confirm that the following items are present in the figure legend, table legend, main text, or Methods section.

n/a	Confirmed	
☐	☒	The exact sample size (*n*) for each experimental group/condition, given as a discrete number and unit of measurement
☐	☒	A statement on whether measurements were taken from distinct samples or whether the same sample was measured repeatedly
☐	☒	The statistical test(s) used AND whether they are one- or two-sided<br>*Only common tests should be described solely by name; describe more complex techniques in the Methods section.*
☒	☐	A description of all covariates tested
☐	☒	A description of any assumptions or corrections, such as tests of normality and adjustment for multiple comparisons
☐	☒	A full description of the statistical parameters including central tendency (e.g. means) or other basic estimates (e.g. regression coefficient) AND variation (e.g. standard deviation) or associated estimates of uncertainty (e.g. confidence intervals)
☐	☒	For null hypothesis testing, the test statistic (e.g. *F*, *t*, *r*) with confidence intervals, effect sizes, degrees of freedom and *P* value noted<br>*Give P values as exact values whenever suitable.*
☒	☐	For Bayesian analysis, information on the choice of priors and Markov chain Monte Carlo settings
☒	☐	For hierarchical and complex designs, identification of the appropriate level for tests and full reporting of outcomes
☒	☐	Estimates of effect sizes (e.g. Cohen's *d*, Pearson's *r*), indicating how they were calculated

Our web collection on statistics for biologists contains articles on many of the points above.

Software and code

Policy information about availability of computer code

Data collection	Data collected in assay development and test accuracy was hosted in Microsoft Excel. Data collected in the household study was hosted using REDCap (Research Electronic Data CAPture tools) and exported to Microsoft Excel.
Data analysis	All statistical analyses were performed using the R-studio environment (R, V 4.0.2 and R studio, V 1.2.1073), with the library 'tidyverse' for data manipulation and summary statistics, 'pROC' for ROC analysis and 'binom' for estimating binomial confidence intervals. The libraries 'ggplot2', 'patchwork', 'cowplot' and 'ggstatsplot' were used for data visualisation.

For manuscripts utilizing custom algorithms or software that are central to the research but not yet described in published literature, software must be made available to editors and reviewers. We strongly encourage code deposition in a community repository (e.g. GitHub). See the Nature Portfolio guidelines for submitting code & software for further information.

Data

Policy information about availability of data

All manuscripts must include a data availability statement. This statement should provide the following information, where applicable:

- Accession codes, unique identifiers, or web links for publicly available datasets
- A description of any restrictions on data availability
- For clinical datasets or third party data, please ensure that the statement adheres to our policy

Provide your data availability statement here.

Human research participants

Policy information about studies involving human research participants and Sex and Gender in Research.

Reporting on sex and gender	Sex was determined based on self-reporting for all participants whose saliva samples were tested as part of assay development and evaluation, and field-testing. Sex and gender were not considered in study design. In the source data available for datasets relating to assay evaluation, data exists for a maximum of 163 female participants and 169 male participants.
Population characteristics	Samples tested in assay development and test accuracy were convenience samples donated to the Bristol Biobank. A total of 346 samples were tested from people aged between 1 and 68 years of age, 49.4% were female. In the household study, 67 people provided saliva samples. Household members were aged between 3 and 51 years of age and 52.2% were female.
Recruitment	Samples deposited to the Bristol Biobank are from various studies, therefore there is no control over sampling strategy. Results are limited by suitable samples available for testing. Household members were recruited as part of the CoMMinS study (COVID-19 Mapping and Mitigation in Schools; https://www.commins.org.uk). Household members linked to CoMMinS (either pupils, parents or staff) and who self-identified as SARS-CoV-2 positive (PCR or lateral flow test) could participate. As individuals self-identified and volunteered to take part, there is self-selection bias involved with this sample. It is also likely that due to testing availablity, those who were symptomatic were more likely to self-identify as positive.
Ethics oversight	Whole saliva from healthy donors (pre- and during the COVID-19 pandemic) was obtained via the Bristol BioBank (NHS REC 20/WA/0273) under the use application U-0042. Pre-pandemic (PP) sample cohorts were obtained in two ways. PP cohort 1 samples were collected in Portugal under local Ethics for a specific research study, remaining samples were stored and used for this work under NHS REC 13/NW/0439. PP cohorts 2-5 were collected under further Bristol BioBank deposit applications, and upon study completion these sample sets were deposited into the Bristol BioBank and released to this project under use application U-0042. Saliva samples were collected from household outbreaks during the CoMMinS study under NHS REC 20/HRA/4876.

Note that full information on the approval of the study protocol must also be provided in the manuscript.

Field-specific reporting

Please select the one below that is the best fit for your research. If you are not sure, read the appropriate sections before making your selection.

☒ Life sciences ☐ Behavioural & social sciences ☐ Ecological, evolutionary & environmental sciences

For a reference copy of the document with all sections, see nature.com/documents/nr-reporting-summary-flat.pdf

Life sciences study design

All studies must disclose on these points even when the disclosure is negative.

Sample size	Sample numbers were decided by the availability of samples required to address the study aims, with awareness of MHRA guidance stipulating a requirement of at least 200 confirmed positive cases and 200 confirmed negative cases to estimate ≥98% sensitivity and ≥98% specificity.
Data exclusions	Samples with volumes too low to assay were excluded from ROC analysis. Four households were excluded in the household study: 3 ineligible and 1 did not provide samples.
Replication	Saliva samples were assayed in duplicate and assay reproducibility was assessed by calculating the coefficient of variation for controls tested in duplicate on the same plate (intra-assay variation) and between plates (inter-assay variation).
Randomization	In test accuracy, samples not assayed as part of development were randomised to the threshold set so that 50% of total cases and 50% of total controls appeared in threshold and validation sets. Stratified random sampling considered the following strata and the number of samples randomly sampled from each stratum to the threshold set: asymptomatic PCR-confirmed (n=4); symptomatic PCR-confirmed (n=12); adult pre-pandemic (n=22) and child pre-pandemic (n=61).
Blinding	Clinical information and index test results were not available to the assessors of the reference standard. This was facilitated by assaying validation samples in a blinded fashion.

Reporting for specific materials, systems and methods

We require information from authors about some types of materials, experimental systems and methods used in many studies. Here, indicate whether each material, system or method listed is relevant to your study. If you are not sure if a list item applies to your research, read the appropriate section before selecting a response.

Materials & experimental systems

n/a	Involved in the study
☐	☒ Antibodies
☒	☐ Eukaryotic cell lines
☒	☐ Palaeontology and archaeology
☒	☐ Animals and other organisms
☒	☐ Clinical data
☒	☐ Dual use research of concern

Methods

n/a	Involved in the study
☒	☐ ChIP-seq
☒	☐ Flow cytometry
☒	☐ MRI-based neuroimaging

Antibodies

Antibodies used

Goat anti-human IgG-HRP antibody; Southern Biotech; Cat#2040-05; RRID: AB_2795644.
Goat anti-human IgA (a-chain-specific)-peroxidase antibody; Sigma-Aldrich; Cat#A0295; RRID: AB_257876

Validation

Anti-human IgG-HRP reacts with the heavy chain of human IgG. It is developed in goat using purified human IgG as the immunogen. Following cross-adsorption it does not react with human IgM or IgA but may react with IgG from other species. It is purified by affinity chromatography on human IgG covalently linked to agarose (https://resources.southernbiotech.com/techbul/2040.pdf).

Anti-human IgA (alpha-chain specific) is developed in goat using purified human IgA as the immunogen. Specificity of the Peroxidase Conjugated Anti-Human IgA is determined by Enzyme Linked Immunosorbent Assay (ELISA). The conjugate is specific for human IgA when tested against human IgA, IgG, IgM, Bence Jones kappa and lambda myeloma proteins. Identity and purity of the antibody is established by immunoelectrophoresis (IEP), prior to conjugation. Electrophoresis of the antibody preparation followed by diffusion versus anti-goat IgG and anti-goat whole serum results in single arcs of precipitation. (https://www.sigmaaldrich.com/deepweb/assets/sigmaaldrich/product/documents/174/187/a7032dat.pdf)

Antibodies were prepared according to manufacturers guidelines and frozen in working aliquots.
